# Search for schizophrenia and bipolar biotypes using functional network properties

**DOI:** 10.1002/brb3.2415

**Published:** 2021-11-10

**Authors:** Inés Fernández‐Linsenbarth, Álvaro Planchuelo‐Gómez, Rosa M. Beño‐Ruiz‐de‐la‐Sierra, Alvaro Díez, Antonio Arjona, Adela Pérez, Alberto Rodríguez‐Lorenzana, Pilar del Valle, Rodrigo de Luis‐García, Guido Mascialino, Pedro Holgado‐Madera, Rafael Segarra‐Echevarría, Javier Gomez‐Pilar, Pablo Núñez, Berta Bote‐Boneaechea, Antonio Zambrana‐Gómez, Alejandro Roig‐Herrero, Vicente Molina

**Affiliations:** ^1^ Psychiatry Department, School of Medicine University of Valladolid Valladolid Spain; ^2^ Imaging Processing Laboratory University of Valladolid Valladolid Spain; ^3^ Psychiatry Service Clinical Hospital of Valladolid Valladolid Spain; ^4^ School of Psychology Universidad de Las Américas Quito Ecuador; ^5^ Psychiatry Service Doce de Octubre University Hospital Madrid Spain; ^6^ Psychiatry Service Cruces Hospital Bilbao Spain; ^7^ Biomedical Engineering Group University of Valladolid Valladolid Spain; ^8^ Psychiatry Service University Hospital of Salamanca Salamanca Spain

**Keywords:** biotypes, bipolar disorder, diffusion, electroencephalogram, network, schizophrenia

## Abstract

**Introduction:**

Recent studies support the identification of valid subtypes within schizophrenia and bipolar disorder using cluster analysis. Our aim was to identify meaningful biotypes of psychosis based on network properties of the electroencephalogram. We hypothesized that these parameters would be more altered in a subgroup of patients also characterized by more severe deficits in other clinical, cognitive, and biological measurements.

**Methods:**

A clustering analysis was performed using the electroencephalogram‐based network parameters derived from graph‐theory obtained during a P300 task of 137 schizophrenia (of them, 35 first episodes) and 46 bipolar patients. Both prestimulus and modulation of the electroencephalogram were included in the analysis. Demographic, clinical, cognitive, structural cerebral data, and the modulation of the spectral entropy of the electroencephalogram were compared between clusters. Data from 158 healthy controls were included for further comparisons.

**Results:**

We identified two clusters of patients. One cluster presented higher prestimulus connectivity strength, clustering coefficient, path‐length, and lower small‐world index compared to controls. The modulation of clustering coefficient and path‐length parameters was smaller in the former cluster, which also showed an altered structural connectivity network and a widespread cortical thinning. The other cluster of patients did not show significant differences with controls in the functional network properties. No significant differences were found between patients´ clusters in first episodes and bipolar proportions, symptoms scores, cognitive performance, or spectral entropy modulation.

**Conclusion:**

These data support the existence of a subgroup within psychosis with altered global properties of functional and structural connectivity.

## Graphical abstract

In this study, our aim was to contribute to a better understanding of the biological heterogeneity in psychoses. For this purpose, we conducted a cluster analysis to identify patients’ subgroups based on the characteristics of their functional network, assessed wit electroencephalogram (EEG) data and methods derived from graph‐theory. Our cluster analysis yielded a two‐cluster solution, where one cluster showed significant alterations in their functional network and a significantly altered structural connectivity network with a widespread cortical thinning, and the other cluster showed a normal functional network.

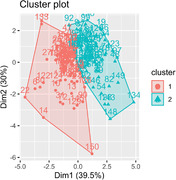

## 1 INTRODUCTION

Schizophrenia may include a heterogeneous population with diverse cerebral alterations that may underlie its clinical variability. Several lines of evidence support this contention. A meta‐analysis showed increased variability within schizophrenia in relevant structural measurements (Brugger & Howes, 2017). Moreover, structural magnetic resonance imaging (MRI) data could discriminate subgroups within schizophrenia with different clinical and biological characteristics (Lubeiro et al., 2016; Planchuelo‐Gómez et al., 2020). Specifically, the existence of a group within the schizophrenia syndrome characterized by higher cortical curvature and lower cortical thickness was revealed (Lubeiro et al., 2016). Moreover, a cluster could be found in schizophrenia and bipolar disorder patients characterized by global cortical thinning associated with cognitive deficits (Planchuelo‐Gómez et al., 2020). Neurophysiological data were also found to be different between treatment‐resistant (TR) and non‐TR schizophrenia patients (Molina et al., 2008). These differences included greater clinical severity in TR patients and a more severe profile of alterations in cerebral anatomical and electrophysiological parameters. Deficit and nondeficit schizophrenia patients showed different structural network properties (Wheeler et al., 2015). Furthermore, white matter abnormalities discriminated between first‐episode (FE) patients with or without severe negative symptoms (Sun et al., 2015). Attending to studies of mixed diagnostic groups of psychosis, another study defined three biotypes cutting across the schizophrenia and schizoaffective and bipolar diagnoses based on cognitive, neuroanatomical, and neurophysiological data (Clementz et al., 2016), highlighting the existence of a subgroup characterized by worse cognition and widespread gray matter (GM) deficits. Furthermore, another study revealed a subgroup of schizophrenia patients with widespread volumetric reductions and worse cognitive deficits (Weinberg et al., 2016). Finally, a recent study aimed at identifying subgroups within schizophrenia and bipolar disorder patients based on their neurocognitive profile revealed the existence of a cognitive severely impaired group which showed higher symptom scores, a hypersynchronic basal connectivity state, and lower fractional anisotropy of frontal tracts (Fernández‐Linsenbarth et al., 2021). Identifying biotypes in psychosis such as schizophrenia and bipolar disorder may contribute to consider them not as homogeneous entities but as syndromes, that is, a collection of symptoms and signs that may have different substrates. Thus, studies based on this consideration of the likely existing heterogeneity in psychosis could contribute to a better understanding of their neurobiological underpinnings, biomarkers definition, and the development of personalized treatments.

The use of cerebral parameters related to the mental functions could be useful in searching pathophysiologically meaningful biotypes within psychosis. Although the cerebral substrates of mental activity are incompletely understood, some facts can be reasonably assumed and may be useful for the purpose of exploring the presence of those biotypes. Mental activity is likely based on the fast‐evolving synchronization of neural assemblies distributed across the brain (Buzsáki & Draguhn, 2004; Varela et al., 2001). The electroencephalogram (EEG) reflects the bioelectrical signal resulting from such synchronization; hence, EEG can be a useful tool for the analysis of the substrates of mental functions. One advantage of the EEG is its high temporal resolution, due to the swiftly evolving synchrony of neural assemblies underlying cognition (Dehaene & Changeux, 2011; Uhlhaas & Singer, 2010). Moreover, higher mental functions such as those altered in schizophrenia likely implicate many cerebral regions, supporting the consideration of global cerebral networks rather than single‐electrode local measurements in analyses aimed at studying possible subtypes of psychosis. The implementation of functional networks assessments based on EEG and graph‐theory measurements offers a tool for such studies, as previously shown (Cea‐Cañas et al., 2020; Gomez‐Pilar, de Luis‐García, Lubeiro, de la Red, et al., 2018; Gomez‐Pilar, de Luis‐García, Lubeiro, de Uribe, et al., 2018).

In the present study, our aim is to explore the possibility of discriminating meaningful biotypes within psychosis based on the global network properties of the EEG and their modulation during cognitive activity. In previous studies, we have shown significant differences in these network properties between schizophrenia patients and controls (Gomez‐Pilar, de Luis‐García, Lubeiro, de la Red, et al., 2018) and between schizophrenia and bipolar patients (Cea‐Cañas et al., 2020). The hypothesis here is that these network properties would be more severely altered in a subgroup of patients also characterized by alterations in other biological, cognitive, and/or clinical measurements. We included bipolar patients due to the clinical and genetic overlapping between these syndromes, considering that these patients are included within the psychosis spectrum. Moreover, in order to discard a major effect of chronicity in the results, we also included first episode patients thus being able to carry out repeated comparisons of these patients within possible resulting subgroups.

## 2 MATERIALS AND METHODS

### 2.1 Participants

Our sample included 183 patients, 102 with chronic schizophrenia, 35 with FE schizophrenia, and 46 with type I bipolar disorder (BD; of them 36 with psychotic features). Patients were diagnosed by one of the experienced psychiatrists in the group according to the criteria of the Diagnostic and Statistical Manual of Mental Disorders 5th edition, considering current mental state, clinical records, and relatives’ information. We also included 158 healthy controls (HC) to compare the cognitive and biological characteristics of the resulting subgroups. All subjects underwent clinical, cognitive, and EEG evaluation. For the assessment of other biological properties, MRI data, including structural and fractional anisotropy (FA) data, were collected in 60 patients and 28 HC. Social cognition data were available for 80 patients and 34 controls.

This sample mostly overlaps (89 patients and 34 HC) with one from a previous report where we searched for MRI‐based clusters in schizophrenia and BD (Planchuelo‐Gómez et al., 2020). Most cases were also included in an assessment of biological differences between patients characterized by their cognitive profile (Fernández‐Linsenbarth et al., 2021).

Exclusion criteria were (a) intelligence quotient under 70; (b) present or past substance dependence (excluding caffeine and nicotine); (c) head trauma with loss of consciousness; (d) neurological or mental primary diagnosis different from schizophrenia or bipolar disorder (for patients); (e) any current neurological or psychiatric diagnosis (for HC); and (f) any other treatment affecting central nervous system. All participants provided written informed consent. The local ethics committee endorsed the study. This work complies with the ethical standards of the Helsinki Declaration, revised in 2008.

### 2.2 Symptoms assessment

Symptoms were scored with the Positive and Negative Syndrome Scale (PANSS) (Kay et al., 1987) and the Brief Assessment of Negative Symptoms Scale (BNSS) (Kirkpatrick et al., 2011).

### 2.3 Cognitive assessment

Cognition was assessed using the Spanish version of the Brief Assessment of Cognition in Schizophrenia (BACS) (Segarra et al., 2011), including performance in verbal memory, working memory, motor speed, verbal fluency, attention and processing speed, and problem solving, and the Wisconsin Card Sorting Test (WCST; percentage of perseverative errors) (Chelune & Baer, 1986). Global Intelligence Quotient (IQ) was evaluated with the Spanish version of the Wechsler Adult Intelligence Scale III (Fuentes Durá et al., 2010). Social cognition was assessed with the Mayer, Caruso, and Salovey Emotional Intelligence scale (MSCEIT) (Mayer et al., 2003).

### 2.4 EEG data

#### 2.4.1 EEG data acquisition

**FIGURE 1:**
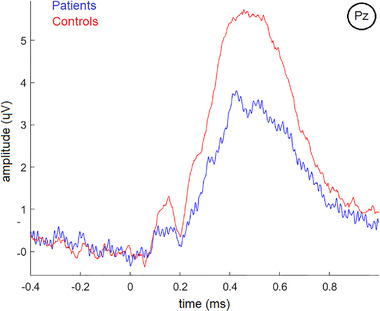
P300 waves for healthy controls (red line) and patients (blue line) on the PZ (midline parietal) electrode for ‘‘attended’’ target tones

**FIGURE 2:**
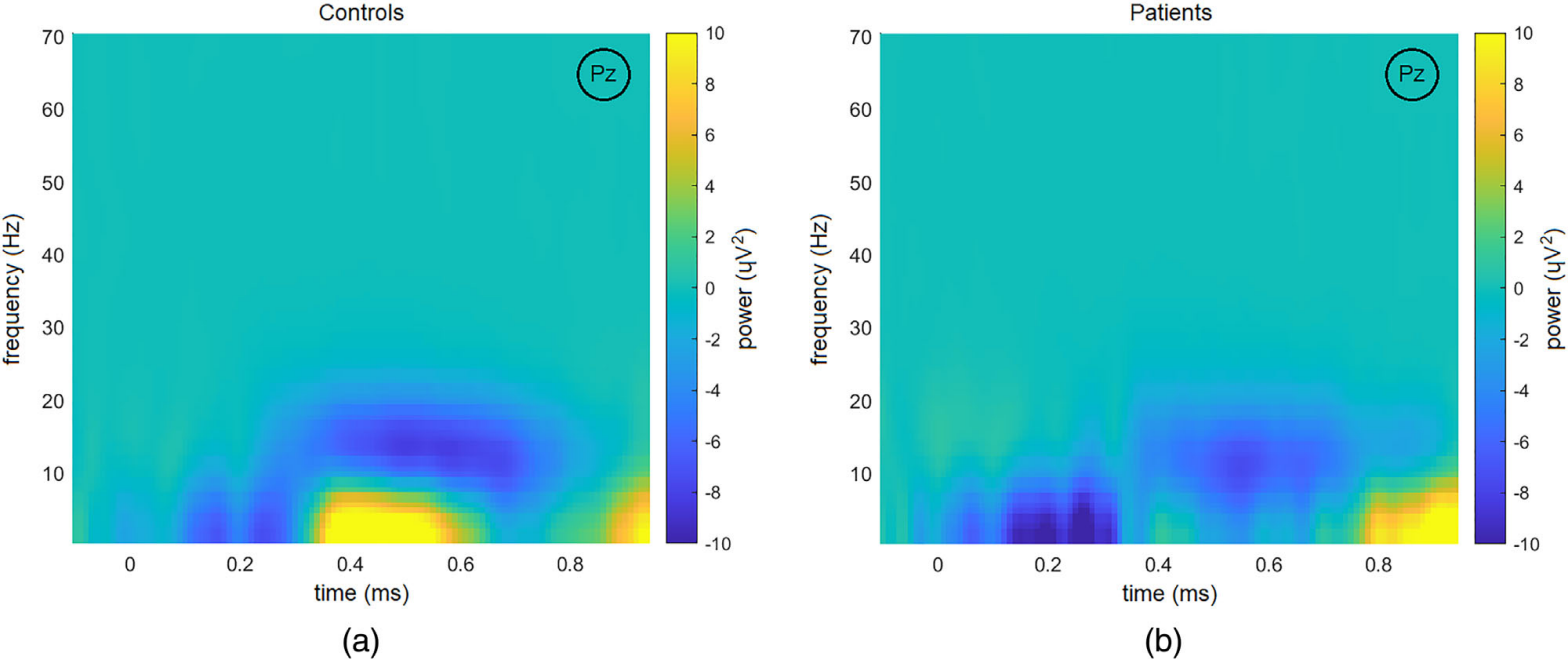
Time‐frequency‐power representations for ‘‘attended’’ target tones on the PZ (midline parietal) electrode for healthy controls (left) and patients (right)

EEG data were recorded during an auditory oddball task from a 32‐channel system (Brain Vision [Brain Products GmbH]) following the international 10–10 system. The auditory oddball three‐condition paradigm presented 600 random stimuli: target (500 Hz tone, probability of 0.2), distractor (1000 Hz tone, probability of 0.2), and standard (2000 Hz tone, probability of 0.6). Each tone lasted 50 ms and comprised a rise and fall time of 5 ms with an intensity of 90 decibels. The interstimulus interval randomly jittered between 1.16 and 1.44 s. Participants were asked to keep their eyes closed and to press a button upon hearing target tones. Target tones were considered ‘‘attended’’ when followed by a button press. Only ‘‘attended’’ target tones were considered for further analysis. Alertness differences were controlled for by comparing the accuracies of the target response. Representative P300 waves for HC and patients are shown in Figure 1 for the ‘‘attended’’ target tones. Time‐frequency‐power representations for the ‘‘attended’’ target tones for HC and patients can be found in Figure 2. Both figures illustrate data after preprocessing, artifact and baseline corrections, and grand‐averaging. More details of EEG data acquisition and preprocessing can be found in the Supporting Information.

#### 2.4.2 EEG‐based brain graphs and connectivity strength calculation

In the construction of EEG‐based brain graphs, network nodes are a mathematical representation of the EEG electrodes, whereas the values of the network edges are calculated from the neural coupling between each pair of electrodes (Stam & van Straaten, 2012). This coupling can be estimated with different methods. Here, we selected the phase‐locking value (PLV) across successive trials (Lachaux et al., 1999), which is sensitive to low‐amplitude oscillatory EEG components (Spencer et al., 2003) in addition to nonlinearities (van Diessen et al., 2015).

The PLV, in turn, can be computed using different methodologies; we used the continuous wavelet transform (CWT) using the convolution of each trial with a scaled and translated version of the complex Morlet wavelet. Thereby, the phase information from each trial is computed (Bob et al., 2008) considering cones of influence to remove edge effects (Torrence & Compo, 1998).

Applying the CWT approach for the performance of filter and phase extraction in one operation, the PLV between two signals, *x*(*t*) and *y*(*t*), was obtained evaluating the variability of the phase difference across successive trials (Gomez‐Pilar, de Luis‐García, Lubeiro, de Uribe, et al., 2018; Lachaux et al., 1999):

(1) $$\mathrm{PL}V_{xy}\;\left(k,s\right)=\frac{1}{N_{t}}\;\left|\sum_{n\;=\;1}^{N_{t}}e^{\Delta \varphi_{xy}\left(k,s,n\right)}\right|,$$

where *N_t_* is the number of trials, Δ*φ_xy_* is the instantaneous phase difference between the signals *x* and *y*, *k* is the time interval, and *s* is the scaling factor of the mother wavelet.

We generated functional connectivity matrices using the PLV values. Due to the fact that no threshold was applied, these connectivity matrices ranged between 0 and 1; 0 was obtained when two signals had no synchronization, and 1 was obtained when two signals were perfectly synchronized.

We selected two windows from the EEG signal: (i) the prestimulus window, which corresponded to a period of expectation before the stimulus onset from −300 ms to the stimulus onset and (ii) the response window, which is related to the P3b response (150–450 ms after the stimulus onset). Thus, the prestimulus window is located during task performance and is completely different from resting state. This procedure was applied both for the EEG theta band (4–8 Hz) and the global band (1–70 Hz) in which higher values of prestimulus connectivity strength (CS) have been reported in previous studies in schizophrenia (Gomez‐Pilar, de Luis‐García, Lubeiro, de la Red, et al., 2018; Gomez‐Pilar, de Luis‐García, Lubeiro, de Uribe, et al., 2018).

#### 2.4.3 Graph parameters

Once the functional connectivity matrices were obtained, the resulting matrices were analyzed by means of different parameters from graph‐theory field in order to characterize global connectivity properties of the brain network. Specifically, the present study focused on four parameters of the brain network: segregation (clustering coefficient [CLC]), integration (path length [PL]), small‐world index (SW), and CS (Gomez‐Pilar, de Luis‐García, Lubeiro, de la Red, et al., 2018). These parameters were computed in two windows: prestimulus (300 ms before stimulus onset) and response (150–450 ms from the stimulus onset, centered around the P300 peak). The corresponding difference between the response and prestimulus window was considered as the modulation value, measuring the degree of change of graph parameters across time, that is, chronnectomics. Prestimulus and modulation network values were used in clustering analysis. Complete details of the graph parameters calculations can be found in the Supporting Information.

It is important to note here the bias introduced by volume conduction effects in EEG studies (Brunner et al., 2016), particularly in approaches involving connectivity metrics. These effects hamper the estimation of the connectivity between the real activity sources. Probably the most used technique to tackle this problem is to perform a source analysis, that is, to solve the inverse problem. These approaches, such as low‐resolution tomography (LORETA), are able to identify activity sources from which connectivity metrics can be computed. However, they are not completely bias‐free, and the inverse solution problem remains unsolved, especially for nonhigh‐density EEG (e.g., Hassan et al., 2014; Michel et al., 2004). A well‐known alternative strategy to minimize field spread is based on the assumption that volume conduction affects the connectivity in a similar way in two different experimental contrasts, such as the prestimulus and response conditions (Bastos & Schoffelen, 2016). Therefore, the comparison between these two conditions, as has been done in this study, reduces volume conduction effects.

#### 2.4.4 Spectral entropy

Furthermore, in previous studies, we identified a deficit of brain activity modulation with cognition in schizophrenia patients during a P300 task using the spectral entropy (SE) parameter (Bachiller et al., 2014; Gomez‐Pilar, de Luis‐García, Lubeiro, de la Red, et al., 2018; Molina et al., 2018) (see the Supporting Information). SE modulation was also computed as the SE difference between response and prestimulus windows (Gomez‐Pilar, de Luis‐García, Lubeiro, de Uribe, et al., 2018), providing a measure of the degree of the signal regularity change across time. Since a decrease on SE from prestimulus to response has been robustly observed in HC, normal SE modulation is expected to be expressed in negative values (Bachiller et al., 2014; Gomez‐Pilar, de Luis‐García, Lubeiro, de la Red, et al., 2018; Molina et al., 2018).

### 2.5 Structural data

The structural data were based on the assessment of GM morphometry from T1‐weighted data and structural connectivity from diffusion‐weighted MRI data. MRI acquisition details can be found in the Supporting Information. The acquisition parameters were the same employed previously to characterize psychosis subgroups from MRI data (Planchuelo‐Gómez et al., 2020).

#### 2.5.1 MRI processing

Using the T1‐weighted images, the segmentation pipeline from FreeSurfer (http://surfer.nmr.mgh.harvard.edu) version 6.0.0 was employed for the automatic cortical parcellation of GM regions (Dale et al., 1999). The average cortical thickness and subcortical GM volume were extracted from regions included in the Desikan–Killiany atlas (Desikan et al., 2006). We restricted our analysis to 14 bilateral cortical regions (Table S1). Moreover, we calculated the GM volume of the hippocampus, thalamus, caudate, putamen, and pallidum, as in our previous work (Lubeiro et al., 2016).

#### 2.5.2 Diffusion tensor imaging data

From the diffusion tensor imaging (DTI), the FA in connections between pairs of regions was assessed, following the processing pipeline described in Lubeiro et al. (2017). Anatomically constrained tractography was obtained using the diffusion‐weighted data (Jenkinson et al., 2012), considering the FA as the structural connectivity metric of interest. The evaluated connections from the tractography were focused on regions from the prefrontal cortex (rostral middle frontal and superior frontal gyri) and the limbic system (entorhinal cortex, parahippocampal gyrus, and hippocampus). Connections in which null values were found in a third (or more) of the subjects were discarded. A total of 46 homolateral connections were analyzed.

### 2.6 Cluster extraction

Data were divided in main and replication datasets, according to the recruitment center. The former included 80 patients with chronic schizophrenia, 35 patients with FE, and 34 patients with BD. The latter was composed of 22 patients with chronic schizophrenia and 12 patients with BD. The EEG parameters employed in the cluster extraction were the PL, the SW coefficient (the CLC divided by the PL), and the CS, all three in the prestimulus and modulation windows, for a total of six variables included in the clustering analysis. These variables were normalized with *Z*‐scores before proceeding to the clustering process to avoid a bias caused by the difference between the values from each parameter. We decided to employ exclusively EEG network parameters to focus on the functional connectivity differences within psychosis and to avoid the employment of an excessive number of parameters from diverse sources that may cause overfitting. Moreover, as secondary analysis, we evaluated structural connectivity and cognition in the clusters extracted from the EEG network parameters.

In the main dataset, 26 indices were employed to extract the optimal number of clusters (Supporting Information). A large number of indices was employed to avoid the bias that may be produced by the use of a single or few indices. The majority rule (the value obtained with a higher number of indices) was used as the selection criterion. In case of a draw, the lowest number was chosen. This process was implemented with the NbClust package included in R, where further details about this clustering process can be found (Charrad et al., 2014).

For a specific number of clusters according to the majority rule, the subgroups were extracted using the *k*‐means clustering methods and 50 initial random centroids. The centroids with the best silhouette profile were chosen (Rousseeuw, 1987).

We used the Clustering Large Application (CLARA) method to assess the consistency of the *k*‐means clustering (Kaufman & Rousseeuw, 1990). Briefly, the CLARA method is based on the *k*‐medoids method, which is similar to the *k*‐means method using the median instead of the mean values. Here, the dataset was divided into 50 random subsets and the Euclidean distance was used as metric. The classification results were compared with the *k*‐means clusters.

To determine the most important features characterizing the subgroups, a linear discriminant function was computed. A jackknife procedure was employed to test the classification accuracy from the discriminant function (Severiano et al., 2011). The discriminant scores were separately computed for controls.

In the replication dataset, the previous procedures were also used. The discriminant function obtained with the main dataset was applied to the patients from the replication dataset and the classification results were compared with the *k*‐means clusters.

As secondary analysis, we repeated the clustering process excluding the BD patients, considering that some of them did not have psychosis and the possible bias that they may introduce into the clustering results.

### 2.7 Statistical analysis of the clusters

Chi‐squared and *t*‐tests were used to compare age, sex distribution, parental education level, positive and negative symptoms, illness duration, and treatment doses between patients’ clusters.

Analysis of variance (ANOVA) followed by pairwise comparisons with Bonferroni correction was used to compare cognitive performance, SE modulation, structural connective network, and regional thickness between patients’ clusters and controls. The *p*‐values from the global ANOVA tests were corrected for multiple comparisons following the Benjamini–Hochberg false discovery rate procedure, grouping these *p*‐values by sets of comparisons (e.g., EEG graph theory parameters). We repeated these comparisons between FE patients in each cluster using Mann–Whitney *U*‐tests to discard a major effect of chronicity in the results, without further correction for multiple comparisons due to the relatively small sample size.

As in our previous studies (Gomez‐Pilar, de Luis‐García, Lubeiro, de Uribe, et al., 2018), the SE modulation in individual sensors was introduced in a factor analysis and the resulting factor scores were used in subsequent comparisons.

## 3 RESULTS

### 3.1 Cluster solutions

**FIGURE 3:**
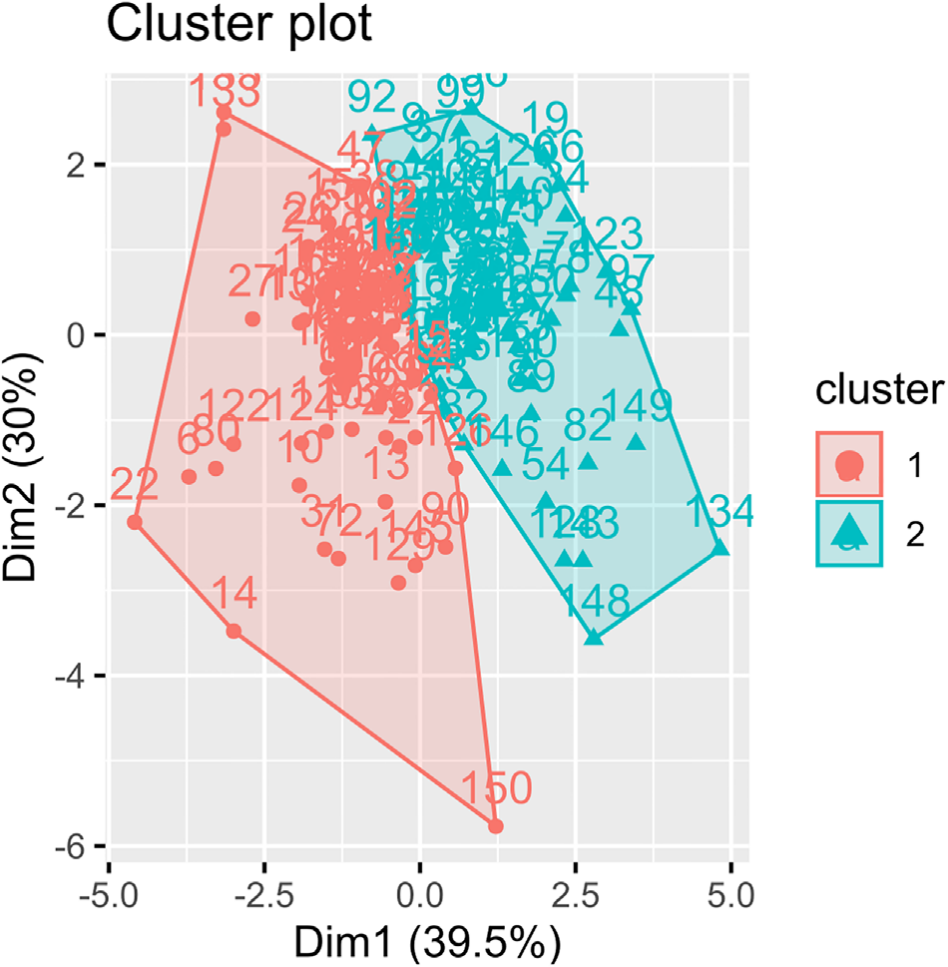
Clusterization of the psychosis subgroups based on electroencephalogram (EEG) graph theory measures in the main dataset. Principal component analysis (PCA) was employed to summarize the scores from the graph theory measures. The horizontal axis represents the first principal component, and the vertical axis the second component. The numbers represent identifiers for each subject

The optimal number of clusters in the main dataset according to the majority rule was determined to be 2. A principal component analysis (PCA) was carried out to summarize the information from the EEG data and illustrate the clusters, but it was not used for clusterization. The reason of not using PCA in the analysis but for illustration is that this method is able to summarize information from diverse variables. Bearing in mind the relatively low number of variables in this analysis, we considered that there was no need to summarize the information and lose part of the original information. Figure 3 shows the cluster plot based on the PCA values, and Figure S1 shows the optimal number of clusters according to the 26 indices in the main dataset. The corresponding results in the replication dataset can be observed in Figures S2 and S3.

Cluster composition was as follows:

- Cluster 1 (C1): 42 chronic schizophrenia, 17 FE schizophrenia, and 16 BD patients from the main dataset and 11 chronic schizophrenia and eight BD patients from the replication dataset;
- Cluster 2 (C2): 38 chronic schizophrenia, 18 FE schizophrenia, and 18 BD patients from the main dataset, and 11 chronic schizophrenia and four BD patients from the replication dataset.

There were no significant differences in patients’ diagnoses distribution between clusters, considering the subjects from both datasets together (*χ* ^2^ = 0.136, df = 2, *p* = 0.93). There were no significant differences between clusters in age, illness duration, or sex distribution.

Compared to the *k*‐means classification of the main dataset, the CLARA method obtained similar results, with 96.0% of the subjects classified in the same clusters. Table S2 shows the comparison of the clusters obtained with *k*‐means and CLARA.

**FIGURE 4:**
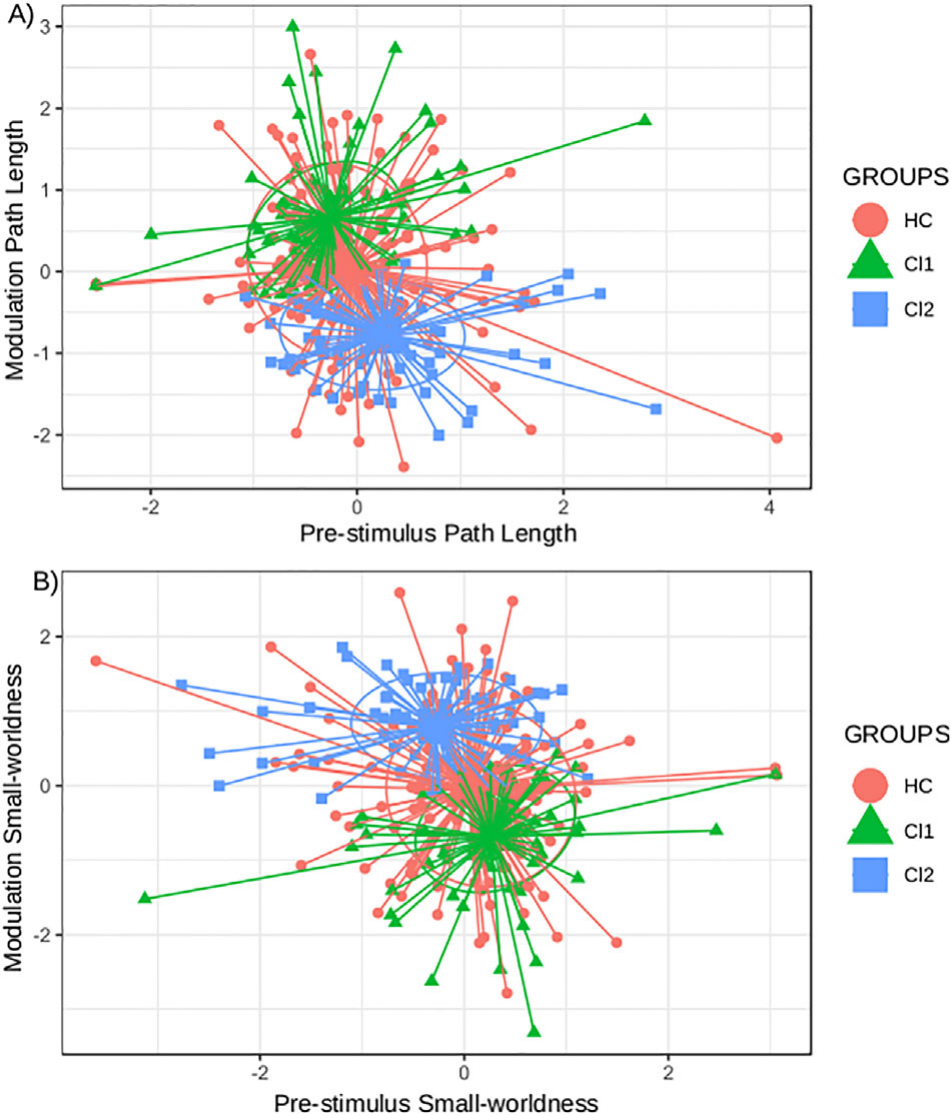
Scatter plot of the distribution of (a) prestimulus path length and modulation and (B) pre‐stimulus small‐worldness and modulation in the identified clusters. Circles represent healthy controls (HC), triangles represent patients from cluster 1 (C1), and squares represent patients from cluster 2 (C2). The ellipsoids have a radius of 1 SD

Regarding the jackknife analysis, 96.0% of accuracy was obtained with respect to the *k*‐means estimation. The classification results and the coefficients of the discriminant function are shown in Tables 1 and S3. In Table 1, it can be observed that the modulation of SW and PL are the factors with the highest weight. Figure 4 depicts the residuals plot with the value of those factors in the identified clusters. More details regarding the differences of the EEG network parameters are shown in Section 3.3. With respect to the classification of the subjects from the replication sample using the discriminant function from the main dataset, 82.4% of the subjects were classified in the same cluster as using the *k*‐means method. The classification of the replication dataset is shown in Table S4.

**TABLE 1:** Coefficients of the linear discriminant function

Variable	Discriminant coefficient
Prestimulus path Length	0.315
Prestimulus connectivity strength	0.063
Modulation path length	1.000
Modulation connectivity strength	−0.361
Prestimulus small‐worldness	−0.223
Modulation small‐worldness	2.408

**FIGURE 5:**
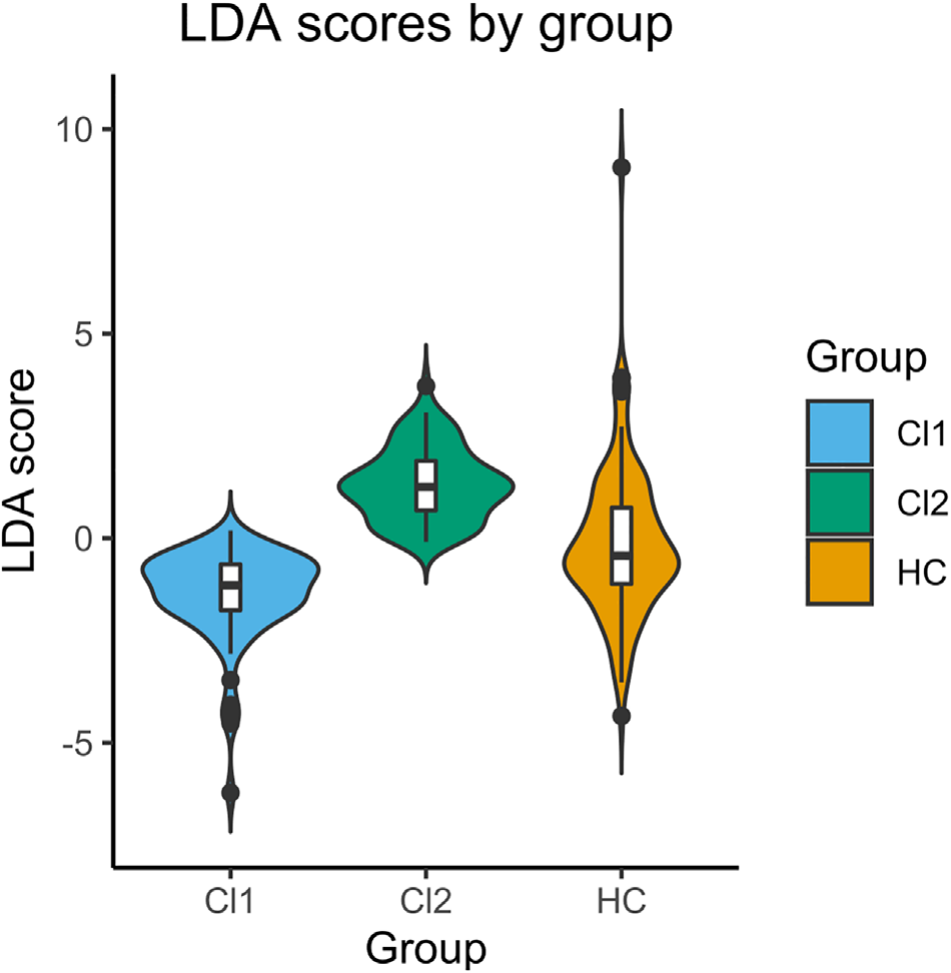
Violin and box plots illustrating the discriminant scores of the patient subgroups and healthy controls from the main dataset. C1, cluster 1; C2, cluster 2; HC, healthy controls; LDA, linear discriminant analysis

The comparison of the discriminant values showed that patients from C2 presented higher scores compared to patients from C1 and controls (*p* < .0001 in both cases), while lower scores were identified in C1 patients with respect to controls (*p* < .0001; Figure 5). In Figure 5, it is worth noting that the outliers could give a wrong impression about the differences between the two clusters groups and controls. The distribution of C2 scores is displaced to considerably higher values compared to controls, and the distribution of C1 scores to lower values. Similar trends were observed with the scores of the replication dataset, but with no statistically significant differences (Figure S4).

Regarding the secondary analysis excluding the BD patients, the extracted results were similar compared to the assessment with the whole database (Figures S5 and S6). One subject (a chronic schizophrenia patient) was excluded because it produced an outlier that biased the clustering results. In C1, the final classification results were 42 chronic schizophrenia and 17 FE patients in the main dataset, and 11 chronic schizophrenia patients in the replication dataset. The FE patients and the patients from the replication dataset were equally classified in comparison with the original analysis, and nine chronic schizophrenia patients who were included in C2 in the original assessment were classified as C1 in this case. In C2, the classification results were 37 chronic schizophrenia patients and 18 FE patients in the main dataset, and 11 chronic schizophrenia patients in the replication dataset. The FE patients and the patients from the replication dataset were equally classified in comparison with the original analysis, and eight chronic schizophrenia patients who were included in C1 in the original assessment were classified as C2 in this case.

With respect to the discriminant function, the values of each factor were similar compared to those from the original analysis, with higher values of the two most important factors (modulation of SW and modulation of PL), and higher relevance of the prestimulus PL. Furthermore, the influence of the prestimulus SW was opposite (positive value) compared to the weight from the original assessment (negative value). These values are shown in Table S5.

### 3.2 Cognitive and clinical comparisons

Current treatment doses were not significantly different between clusters (Table 2).

**TABLE 2:** Clinical, cognitive, and demographic data

	Cluster 1	Cluster 2	Controls
Age	38.59 (11.04)*	39.06 (11.96)*	31.49 (11.33)
Illness duration	6.92 (8.38)	16.01(26.67)	N/A
Parents education (years)	11.07(4.85)*	9.95 (3.11)*	13.60 (4.49)
Verbal memory (BACS)	36.58 (12.51)**	37.50 (9.86)**	51.61 (8.05)
Working memory (BACS)	16.75 (4.97)**	16.98 (4.33)**	21.78 (3.61)
Motor speed (BACS)	60.94 (19.12)**	64.09 (16.59)*	70.57 (17.67)
Verbal fluency (BACS)	18.70 (7.15)*	19.47 (6.60)*	23.61 (10.67)
Performance speed (BACS)	41.21 (14.91)**	43.74 (11.29)**	67.72 (12.75)
Problem solving (BACS)	15.79 (4.92)*	16.34 (3.13)	17.81 (2.75)
%Perseverative errors (WCST)	19.80 (14.51)	16.82 (9.69)**	10.83 (8.83)
Total IQ (WAIS)	91.54 (14.69)**	94.55 (14.21)	113.45 (12.42)
Emotional intelligence (MSCEIT)	96.92 (21.55)**	99.47 (21.17)**	121.90 (11.36)
Positive symptoms (PANSS)	10.57 (3.69)	11.30 (4.91)	N/A
Total negative symptoms (BNSS)	22.84 (17.73)	21.16 (16.21)	N/A
Total symptoms (PANSS)	51.40 (20.34)	48.36 (20.19)	N/A
CPZ equivalents (mg/day)	367.65(348.65)	295.29(212.09)	N/A

*Notes*: Data are shown as mean (SD). All the results from the ANOVA comparisons assessing demographic variables and cognition survive the correction for multiple comparisons.

Abbreviations: BACS, Brief Assessment of Cognition in Schizophrenia; BNSS, Brief Negative Symptom Scale; GEOPTE, Scale for Social Cognition for Psychosis; IQ, Intelligence Quotient; MSCEIT, Mayer‐Salovey‐Caruso Emotional Intelligence Test; PANS, Positive and Negative Syndrome Scale WAIS: Wisconsin Card Sorting Test; WCST, Wechsler Adult Intelligence Scale.

^*^ *p *< .05; ^**^ *p *< .01 in comparison to healthy controls. These *p*‐values are adjusted by corrections for multiple comparisons. There were no significant differences between clusters of patients.

Positive and negative symptoms were not significantly different between C1 and C2 patients, nor between FE patients in these clusters (Table S6).

Cognitive performance according to BACS and WCST was not significantly different between clusters. Global IQ did not differ between clusters. There were no significant differences between C1 and HC in percentage of perseverative errors (WCST) nor between C2 and HC in problem solving and total IQ. For all other cognitive domains, scores were significantly lower in both patients’ clusters as compared to HC (Table 2). When considered alone, FE patients in C2 showed lower performance than FE cases in C1 in working memory (*U* = 55, *z* = −2.15, *p *= .031) and, at trend level, in problem solving (*U* = 61.0, *z* = −1.7964, *p *= .10) (Table S6). There were no significant differences in social cognition scores between clusters.

### 3.3 EEG network parameters

A significant effect of the group was found for all variables of the functional network (4.44 < *F* < 62.48; .01 > *p *> .0001). In the post hoc comparisons, prestimulus CS, CLC, and PL were higher in C2 as compared to C1 and HC. SW was lower in C2 compared to C1 and HC (Table 3).

**TABLE 3:** Prestimulus network values and its modulation with P300 task

	Cluster 1 (*n* = 94)	Cluster 2 (*n* = 89)	Controls (*n* = 158)
Averaged clustering coefficient (CLC)	1.005 (0.003)	1.007 (0.004)**/##	1.005 (0.003)
Characteristic path length (PL)	1.077 (0.022)	1.100 (0.028)**/##	1.083 (0.033)
Connectivity strength (CS)	0.310 (0.050)	0.321 (0.032)**	0.300 (0.033)
CLC modulation	0.001 (0.001)	0.000 (0.001)**/##	0.001 (0.001)
PL modulation	0.007 (0.007)**	−0.006 (0.006)**/##	0.002 (0.009)
CS modulation	0.001 (0.007)	−0.003 (0.009) #	0.000 (0.010)
Small‐world index	0.933 (0.018)**	0.916 (0.021) **/##	0.928 (0.024)
Small‐world modulation	−0.005 (0.005)**	0.005 (0.004) **/##	−0.001(0.006)

*Note*: Data are shown as mean (SD). All the ANOVA results survive the correction for multiple comparisons.

^*^ *p *< .05; ^**^ *p *< .001 as compared to healthy controls.

^#^ *p *< .05; ^##^ *p *< .001 between patients’ clusters.

The previous *p*‐values are adjusted by corrections for multiple comparisons.

Modulation of CLC and PL parameters was smaller in C2 compared to C1 and HC, indicating that these parameters decreased in the active window in C2 but increased in C1 and HC. SW modulation was higher in C2 (where SW value was higher in the response window) than in C1 and HC (where SW was lower in the response window). Modulation of CS was lower in C2, implying that CS increased more in C1 than in C2 (Table 3).

FE patients in C2 showed significantly longer baseline PL (*U* = 75, *z* = 2.39, *p *= .007) and smaller SW values in broadband (*U* = 76, *z* = −2.36, *p *= .018) compared to C1 FE patients. In C2, modulation values of PL (*U* = 4, *z* = −4.83, *p *< .001) and CLC (*U* = 39, *z* = −3.64, *p *< .001) were also smaller than C1 FE patients, and modulation of SW (*U* = 4, *z* = −3.83, *p *< .001) was higher in C2 patients (i.e., its values were higher in the response) (Table S7).

### 3.4 Entropy modulation

As in our previous studies, a single factor summarized most of the variance for entropy modulation, with all sensors contributing positively to that factor. Factor scores for spectral entropy modulation values did not differ between clusters (C1 mean = 0.355, SD = 0.518; C2 mean = 0.194, SD = 0.666), but were significantly more positive in both clusters than in controls (mean = −0.243, SD = 1.185; *F* = 8.59, *p *< .0001), that is, as expected, EEG entropy did not decrease in both patient clusters.

These values were also not significantly different between FE patients in both clusters (*U* = 1272, *z* = −0.95, *p *= .339).

### 3.5 Structural connectivity network

A significant effect of group was found for structural PL (*F* = 3.76, *p* = .027, adjusted‐*p* = .039), SW (*F* = 3.69, *p *= .029, adjusted‐*p* = .039), and CS (*F* = 5.78, *p *= .004, adjusted‐*p* = .016), where post hoc comparisons showed a significantly longer mean PL and smaller SW index in C2 as compared to C1 and HC. Structural connectivity strength was smaller in C2 as compared to HC (Table 4).

**TABLE 4:** Structural connectivity network values

	Cluster 1 (*n* = 30)	Cluster 2 (*n* = 30)	Controls (*n* = 27)
Clustering coefficient	0.995 (0.002)	0.995 (0.003)	0.996 (0.002)
Characteristic path length	1.015 (0.007)	1.019 (0.011)*	1.014 (0.005)
Small‐world index	0.980 (0.007)	0.976 (0.012)*	0.982 (0.006)
Connectivity strength	0.324 (0.035)	0.314 (0.037)**	0.345 (0.030)

*Note*: Data are shown as mean (SD). The ANOVA results with statistically significant results survive the correction for multiple comparisons.

^*^ *p *< .05; ^**^ *p *< .01 as compared to healthy controls. These *p*‐values are adjusted by corrections for multiple comparisons. There were no significant differences between clusters of patients.

### 3.6 Cortical thickness

Significant effects of group were found for bilateral caudal and rostral anterior cingulate cortex, parahippocampal gyrus, pars orbitalis, pars triangularis, precentral cortex, insula, and superior temporal gyrus regions (3.42 < *F* < 9.81; .16 > *p *> .00001). Post hoc comparisons showed a widespread decrease of cortical thickness in C2 as compared to controls (Table S1). With respect to C1, C2 showed a thickness decrease in right caudal anterior cingulate, right cuneus, and right insula. In comparison to HC, C1 patients showed a thinner cortex in a smaller number of regions than C2 (Table S1).

C2 showed a bilateral thalamic volume decrease in comparison to controls, although both comparisons did not survive the correction for multiple comparisons.

Given the small number of FE patients with MRI data, we did not compare structural data in FE patients between clusters.

#### 3.6.1 DISCUSSION

Our results showed two clusters of patients with different functional EEG network patterns across schizophrenia and bipolar syndromes. The EEG network pattern of C1 was similar to that of healthy subjects, while C2 showed larger prestimulus CLC, PL, and CS, and smaller SW values, with decreased modulation of CLC, PL, and CS. The structural connective network showed altered patterns in C2 (with larger PL and smaller CS and SW values). Cortical thickness was regionally decreased in both groups, although this decrease was more widespread in C2. We did not find significant differences in symptoms severity, cognitive performance, illness duration, or antipsychotic doses between clusters.

In our previous results using graph‐theory applied to EEG data in schizophrenia we found several baseline alterations. We reported that schizophrenia patients showed higher CS values at baseline compared to HC (Gomez‐Pilar, de Luis‐García, Lubeiro, de la Red, et al., 2018) and to bipolar patients (Cea‐Cañas et al., 2020). Besides, we also described in schizophrenia patients higher CLC values at baseline (Gomez‐Pilar et al., 2017). These alterations were found in the schizophrenia patients when considered as a whole and could be expected to be more severe in a subgroup of cases such as the cluster 2 in the present study.

Since SW is the CLC/PL ratio, the higher modulation SW values in C2 is possibly consequence of the higher increase of PL in C1 and HC, perhaps due to the involvement of a larger number of cortical regions during task performance. Since response parameters are baseline corrected with respect to the prestimulus condition, a larger increase in PL would diminish SW at response and consequently lead to smaller SW values in this window and thus to negative modulation values of SW in C1 and HC.

Contrary to our expectations, the clusters based on functional network characteristics did not differ in their cognitive performance or symptoms. This may suggest that different cerebral substrates may underpin similar clinical manifestations in different groups, that is, symptoms and cognitive deficits may arise from different substrates. One of such substrates may relate to an alteration of the functional network, but our data suggest that even with a normal functional network other factors may hamper cognition and underlie symptoms, which seems coherent with the data supporting the biological heterogeneity of schizophrenia substrates (Arnedo et al., 2015; Molina & Blanco, 2013; Volk et al., 2012). Our results suggest that an alteration in structural connectivity (only found in C2) may be associated with an altered functional network in only a subset of cases, although we cannot conclude that the former causes the latter. Perhaps the other patients’ symptoms and cognition may be underpinned by a biochemical disbalance not reflected in the functional architecture but that could be caught by other kind of functional analyses. Furthermore, we assessed functional connectivity related to a task, but not resting‐state functional connectivity. The specific behavior associated with a particular task may present different properties compared to resting‐state functional connectivity. It has been reported that task‐based and resting‐state functional connectivity present different network properties (Di et al., 2013), considering additionally the distinct networks for specific tasks and resting‐state conditions. Therefore, structural connectivity, cognition, and clinical symptoms may be more related to resting‐state or other tasks rather than the assessed task‐based functional connectivity of this study.

In support of such possibility, both clusters showed a decreased modulation of their EEG activity during an oddball task as measured with SE. We have described and replicated such a modulation deficit in schizophrenia and bipolar disorder as a possible biomarker for the altered function in this disorder (Molina et al., 2020, 2018). That deficit was associated with cognitive deficits and negative symptoms (Molina et al., 2020, 2018). This could explain why clusters in the present study did not differ in these clinical dimensions, since SE modulation is similar between the clusters extracted in this study.

In a previous study that aimed to explore the existence of clusters based on the neurocognitive profile, we reported that patients with more severe cognitive deficits were also characterized by higher prestimulus CS of the EEG network (Fernández‐Linsenbarth et al., 2021). Thus, we could expect a significant cognitive deficit in C2 with a higher CS, but this was not found. Nevertheless, in that study, both cognitive clusters showed a similar pattern of differences as compared to controls in other neurophysiological data, that is, the same decrease in SE modulation and a smaller CS modulation than HC. No other functional network characteristics were different between the two cognitive clusters identified in that study, where both clusters showed a significant deficit in cognition as in the present one. Probably, depending on the clustering criteria (cognition or EEG), the resulting group correlates may differ slightly, although the global pattern would be that both normal and altered EEG network characteristics at baseline and its modulation may be associated with different degrees of cognitive alteration in schizophrenia and bipolar patients. This could also be consistent with the possibility that altered functional network dynamics are only one of many possible factors leading to symptoms and cognitive impairment in these syndromes.

In the present study, C2 patients also showed altered structural connectivity network properties. In a previous report, we did not find a significant correlation between abnormal values of EEG network parameters and DTI‐based network parameters in schizophrenia (Gomez‐Pilar, de Luis‐García, Lubeiro, de la Red, et al., 2018). This apparent discrepancy may be explained by two factors: that report included 39 schizophrenia patients, while the present one includes 60 patients with EEG and DTI, and we calculated correlations in the global sample, while in the present study we compared values between clusters. The structural connectivity alteration in C2 does not imply that such alteration underlies the corresponding functional network abnormalities.

On the other hand, the regional thinning was more widespread in C2, which is reminiscent of our previous report of two clusters characterized by different patterns of cortical thickness alterations (Planchuelo‐Gómez et al., 2020). There, we did not assess the EEG network; thus, it is possible that both sets of findings converge on the description of a schizophrenia cluster with significant anatomical (cortical thickness and structural connectivity) deficits and an altered functional network. One explanation could be that an anatomically normal schizophrenia would also exist where an altered modulation of EEG activity may appear, and perhaps both alterations contribute to its clinical manifestations.

Interestingly, the results show that clusters did not group themselves based on diagnostic categories. Traditionally, studies have tried to identify the underlying pathophysiological mechanisms of clinical diagnoses, considering them as unitary entities. However, results from data‐driven methodologies, like the one used in this study and the biotype literature, could support the consideration of disorders such as schizophrenia and bipolar disorder as syndromes, including relevant subgroups with different underpinnings. In this line, our results show that certain anatomical and functional brain abnormalities may co‐occur in patients with different diagnoses, and those patients within the same diagnose could present different substrates. This change in the way these disorders are conceived could also shed light on the traditionally lack of consistent replication of cerebral findings in them.

As a future research line, the integration of MRI, cognition, and EEG data should be performed to understand the interactions between structure and function, and their relationship with cognition. Previously, the heterogeneity concerning GM morphometry and cognition has been assessed (Fernández‐Linsenbarth et al., 2021; Planchuelo‐Gómez et al., 2020). The separate evaluation of each area is important to understand the diverse individual mechanisms related to psychosis and the associations between the different paths of psychosis.

To conclude, it is worth noting that our data support the existence of different subgroups within psychosis and may contribute to considering schizophrenia and bipolar disorder not as homogeneous nosologically entities but as syndromes. This notion can also help address the lack of consistent results in previous literature. Finally, the characterization of subgroups could contribute to understanding underlying pathophysiological mechanisms. These mechanisms may be more easily identified through the analyses of biological parameters characterizing subtypes than by comparing schizophrenia patients and controls. This could also raise the possibility of developing personalized treatments based on the most relevant altered underpinnings.

### 3.7 Limitations

Our study has limitations. The first limitation is its sample size. Although a larger sample size would have been desirable, we were able to obtain solid results. Second, biological tests were not available in all subjects. Third, the lack of untreated patients mean that we cannot discard an effect of treatment on EEG values, although we have previously reported nonsignificant effects of antipsychotic, antidepressants, lithium, and benzodiazepines for these values (Molina et al., 2020). Finally, although the volume conduction effects were minimized by comparing two experimental contrast (i.e., EEG modulation), the results should be cautiously interpreted.

## 4 CONCLUSIONS

In conclusion, we found that an abnormal EEG‐based connectivity network is present in approximately half of the patients with schizophrenia and bipolar patients in which significant anatomical changes related to GM cortical thickness and white matter connectivity were also found. These alterations seem independent of chronicity and antipsychotic treatment. EEG network alterations may characterize a biotype across schizophrenia and bipolar diagnoses.

### PEER REVIEW

The peer review history for this article is available at https://publons.com/publon/10.1002/brb3.2415

## Supporting information

Figure S1

Figure S2

Figure S3

Figure S4

Figure S5

Figure S6

Supporting Information

## Data Availability

The datasets that support the findings of this study is available from the corresponding author upon request.
