# Supplementary figures and images for: Search for schizophrenia and bipolar biotypes using functional network properties
Source: Brain Behav. 2021 Nov 10;11(12):e2415. doi: 10.1002/brb3.2415 (PMC8671779; doi:10.1002/brb3.2415)

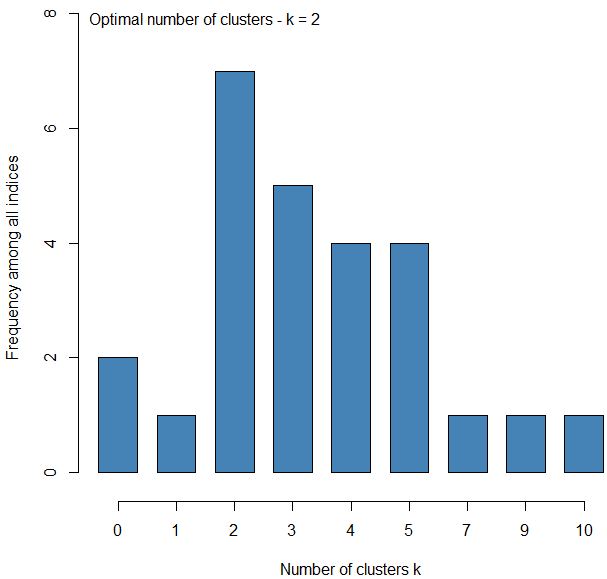

Supplement: Supplementary file 1 — Figure S1 [file BRB3-11-e2415-s007.png]

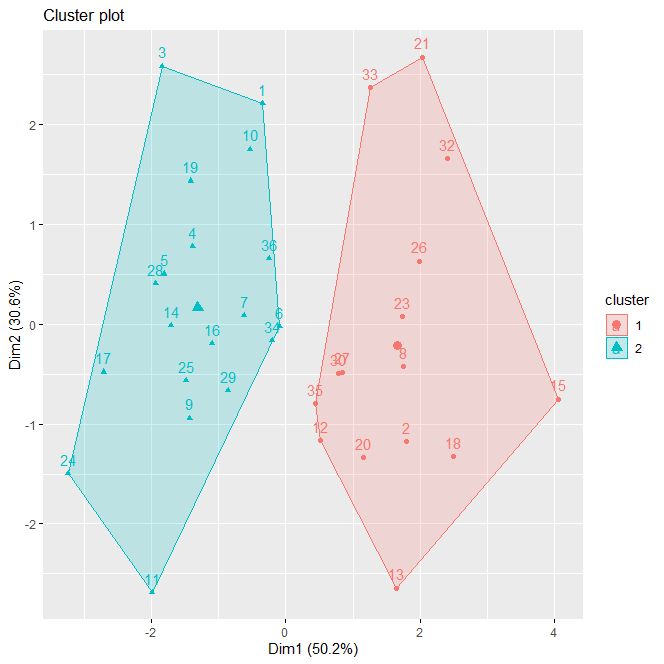

Supplement: Supplementary file 2 — Figure S2 [file BRB3-11-e2415-s006.png]

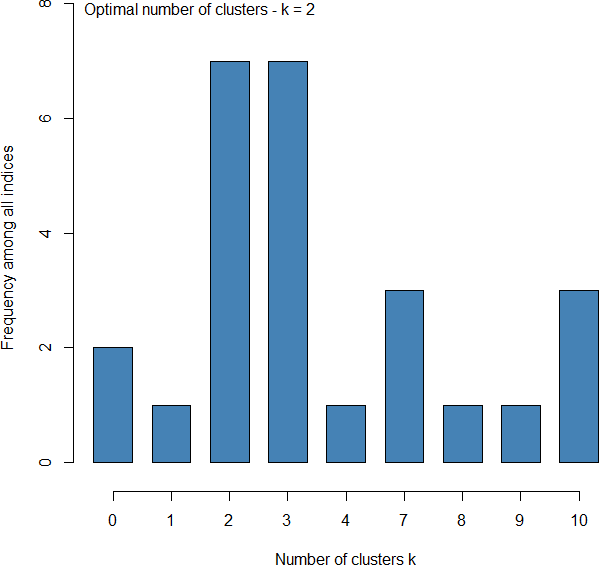

Supplement: Supplementary file 3 — Figure S3 [file BRB3-11-e2415-s005.png]

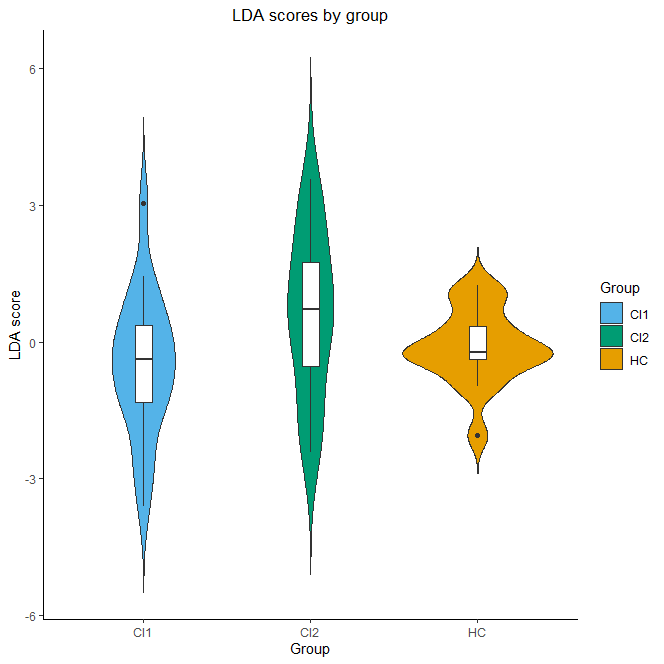

Supplement: Supplementary file 4 — Figure S4 [file BRB3-11-e2415-s002.png]

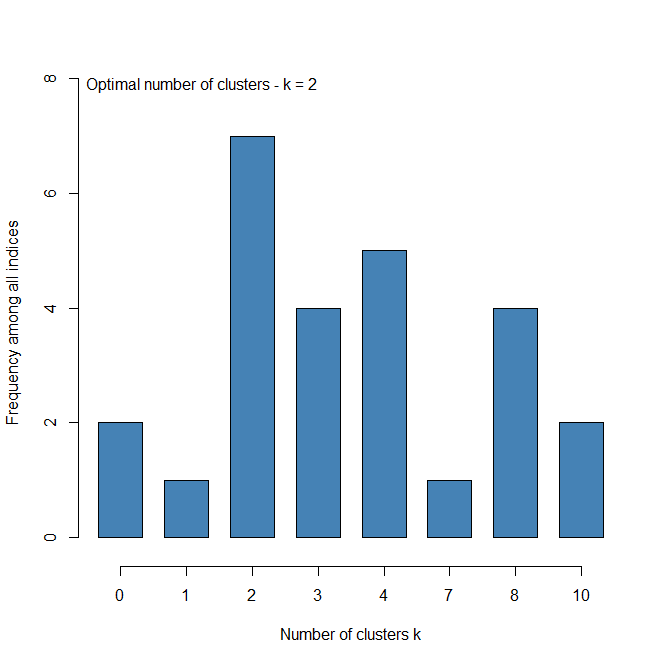

Supplement: Supplementary file 5 — Figure S5 [file BRB3-11-e2415-s001.png]

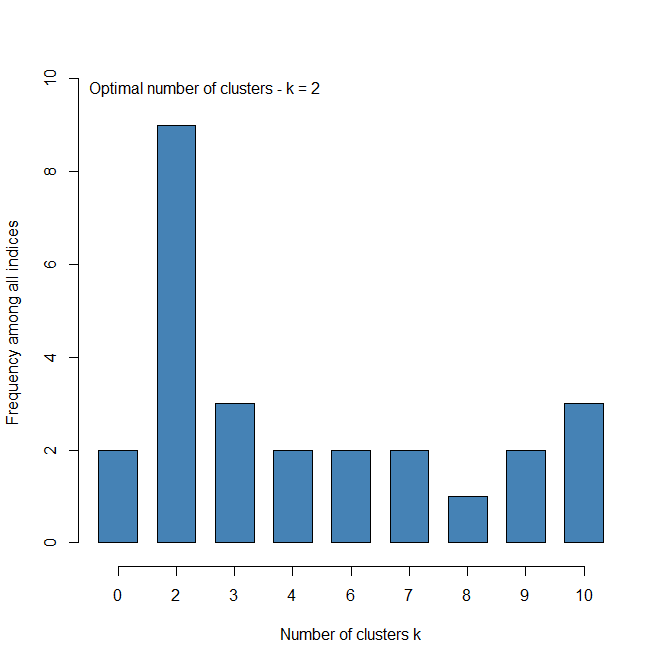

Supplement: Supplementary file 6 — Figure S6 [file BRB3-11-e2415-s003.png]
