# Supplementary material for: Search for schizophrenia and bipolar biotypes using functional network properties
Source: Brain Behav. 2021 Nov 10;11(12):e2415. doi: 10.1002/brb3.2415 (PMC8671779; doi:10.1002/brb3.2415)
Supplement: Supplementary file 7 — Supporting Information [file BRB3-11-e2415-s004.doc]

### **Supplementary Material**

### 1. EEG recording and processing

### 1.1.EEG data acquisition and preprocessing

We recorded the EEG data using a 32-channel EEG system (BrainVision®, Brain Products GmbH). Electrodes were placed at Fp1, Fp2, F7, F3, Fz, F4, F8, FC5, FC1, FCz, FC2, FC6, T7, C3, Cz, C4, T8, CP5, CP1, CP2, CP6, P7, P3, Pz, P4, P8, O1, Oz and O2 following the international 10–10 system; the impedance was kept below 5 kΩ, and the sampling frequency was 500 Hz. The channels were referenced over Cz during acquisition and re-referenced offline to the averaged activity of all of the sensors (Bledowski et al., 2004; Gomez-Pilar, de Luis-García, Lubeiro, de la Red, et al., 2018).

After recording EEG activity during the auditory oddball task the following three-step artifact rejection algorithm was applied to minimize electrooculographic and electromyographic contamination (Bachiller et al., 2014): (i) an independent component analysis (ICA) was performed to discard noisy ICA components; (ii) the signals were divided after ICA reconstruction into trials of 1 s (from 300 ms prior to the stimulus onset to 700 ms after); and (iii) the trials with amplitudes that exceeded an adaptive statistical-based threshold were automatically rejected (Núñez et al., 2017). The signals were band-pass filtered between 1 and 70 Hz, and a 50-Hz notch filter was utilized to remove the power line artifact.

### 1.2.Graph parameters

Once the functional connectivity matrices were obtained, the resulting matrices were analyzed by means of different parameters from graph-theory field. First, the connectivity strength (CS) was computed as follows (Gomez-Pilar, de Luis-García, Lubeiro, de Uribe, et al., 2018):


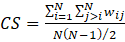
, (1)

where *w_ij_* refers to PLV between nodes *i* and *j*, and *N* is the total number of nodes of the network. Finally, task-related CS modulation was defined as the change of the CS values between the pre-stimulus and the response windows (i.e., CS at the response minus CS at the pre-stimulus windows).

To estimate the network segregation, we rely on the definition of clustering coefficient (CLC) (Rubinov & Sporns, 2010), which, in the case of weighted networks can be generalized as follows:


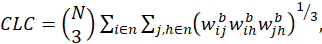
 (2)

where denotes the edge weight between electrodes *i* and *j*.

To quantify the integration of the network, we computed *PL*. It is defined as the average shortest path length between all pairs of nodes in the network (Rubinov & Sporns, 2010):


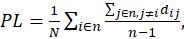
 (3)

where *d_ij_* indicates the minimum distance between electrodes *i* and *j*.

Finally, the ratio between segregation and integration of the network was computed by means of the small-world index (SW). Prior to perform this ratio between CLC and PL it is necessary to remove the dependence with other network parameters, such as the network size or density (Stam et al., 2009). For that purpose, surrogate data were obtained by randomly reshuffling the network connections (Stam et al., 2009). Thus, normalized CLC (*γ*) and normalized PL (*λ*) was computed as follows: (Stam et al., 2009)


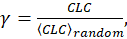
 (4)


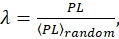
 (5)

Where
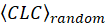
 and
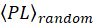
 denote *CLC* and *PL* averaged over an ensemble of 50 surrogate networks, which were computed from a randomization of the original network by reshuffling its connections (Stam et al., 2009). The SW was then computed as their ratio (Rubinov & Sporns, 2010):


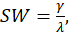
 (6)

### 1.3.Spectral entropy

The spectral entropy (SE) is a measure of the entropy applied over the EEG power spectrum. This means that SE is an estimation of the flatness of the spectral content (Scheeringa et al., 2011). In the time domain, SE can be considered an index of signal irregularity, since it measures how the the frequency of the different oscillators are distributed (Gomez-Pilar, de Luis-García, Lubeiro, de la Red, et al., 2018). In this study, SE was computed from the normalized CWT. The CWT was selected due to its balance between frequency and time resolution (Núñez et al., 2017), which can be set depending on the specific problem. It is possible to directly obtain the wavelet scalogram from the wavelet coefficients as their squared modulus. Then, SE can be defined as follows:


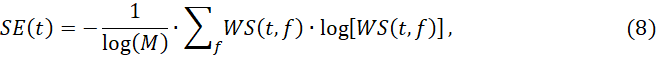
 (7)

where WS is the normalized wavelet scalogram. As for the graph measures, the SE was computed in two windows: prestimulus (300 ms before stimulus to stimulus onset) and response (150 ms to 450 ms from the stimulus onset, centered around the P300 peak). The SE modulation was defined as the change from prestimulus window to the window during the task-related activity.

### **2. MRI acquisition**

In subjects that compose the main dataset, high resolution 3D T1-weighted and diffusion-weighted MRI data were acquired using a Philips Achieva 3T MRI unit (Philips Healthcare, Best, The Netherlands) with a 32-channel head coil. T1-weighted images were acquired with a Turbo Field Echo (TFE) sequence, repetition time (TR) = 8.1 ms, echo time (TE) = 3.7 ms, flip angle = 8º, 256 x 256 matrix size, 1 x 1 x 1 mm^3^ of spatial resolution and 160 slices covering the whole brain. Diffusion-weighted images (DWI) were acquired with TR = 9000 ms, TE = 86 ms, flip angle = 90º, 61 gradient directions, one baseline volume, *b*-value = 1000 s/mm^2^, 128 x 128 matrix size, 2 x 2 x 2 mm^3^ of spatial resolution and 66 axial slices covering the whole brain. The T1-weighted image was obtained in first place, followed by the diffusion-weighted scan in the same session.

### **3. Indices used to extract the optimal number of clusters**

1. Silhouette method.
2. CH index (Calinski and Harabasz)
3. Duda index
4. Pseudot2 index.
5. C-index.
6. Beale index.
7. Cubic Clustering Criterion.
8. PtBiserial index.
9. DB index (from Davies and Boulding).
10. Frey index.
11. Hartigan index.
12. Ratkowsky index.
13. Scott index.
14. Marriot index.
15. Ball index.
16. TraceCovW index.
17. TraceW index.
18. Friedman index.
19. Mcclain index.
20. Rubin index.
21. KL index (from Krzanowski and Lai).
22. Dindex.
23. Dunn index.
24. Hubert index.
25. SDindex.
26. SDbw.

### **Supplementary Figures**


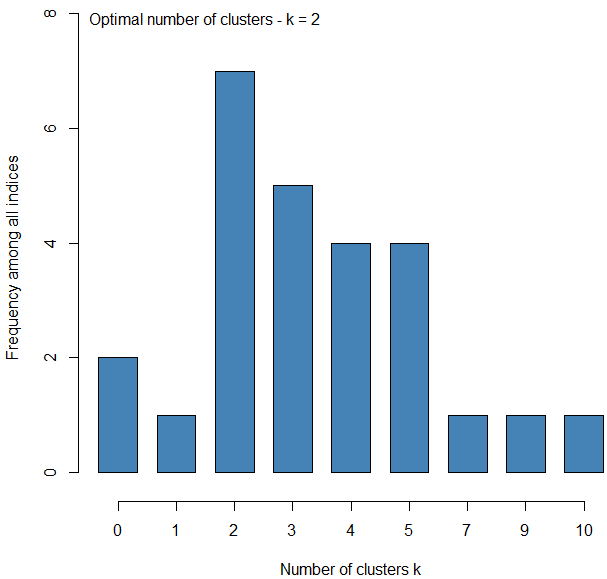


**Figure S1.** Optimal number of clusters for the 26 indices in the main dataset.


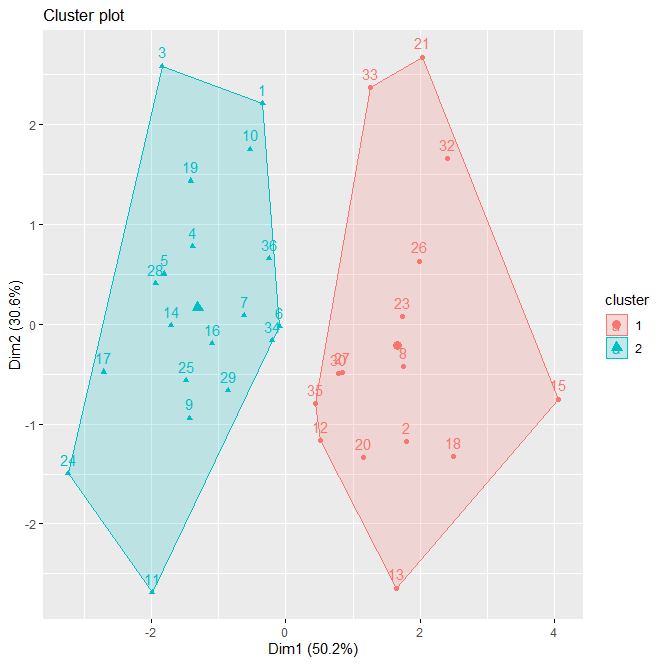


**Figure S2.** Clusterization of the psychosis subgroups based on EEG graph theory measures in the replication dataset. PCA was employed to summarize the scores from the graph theory measures. The horizontal axis represents the first principal component, and the vertical axis the second component. The numbers represent identifiers for each subject.


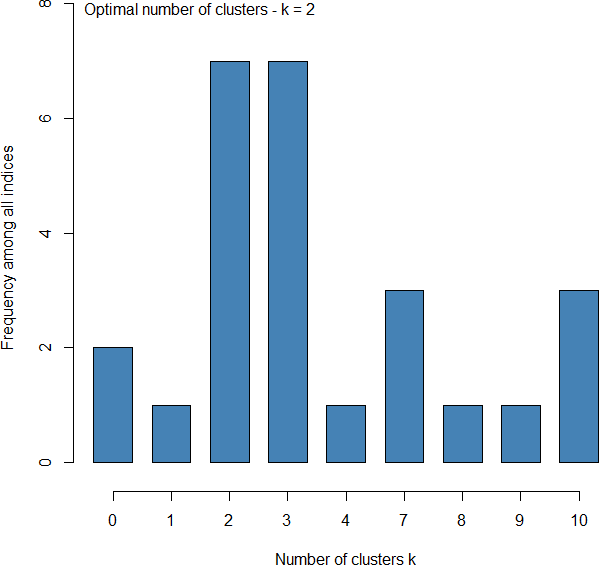


**Figure S3.** Optimal number of clusters for the 26 indices in the replication dataset.


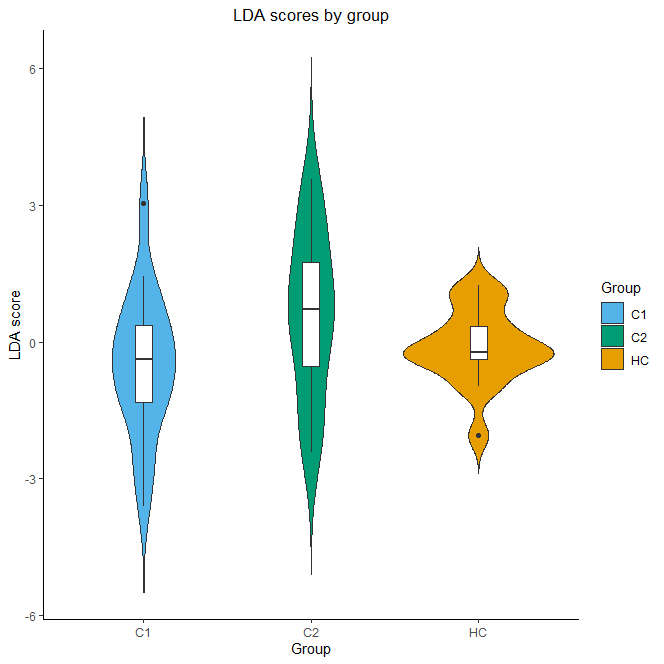


**Figure S4.** Violin and box plots illustrating the discriminant scores of the patient subgroups and healthy controls from the replication dataset. C1 = cluster 1, C2 = cluster 2, HC = healthy controls, LDA = linear discriminant analysis.


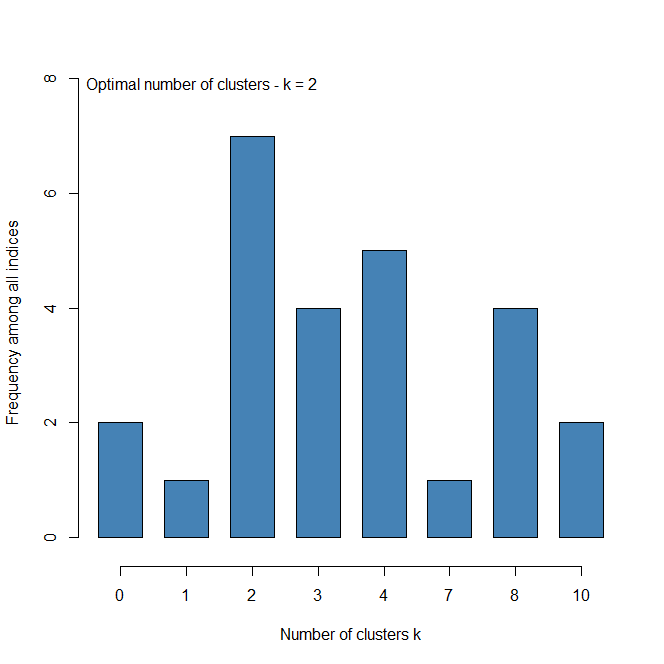


**Figure S5.** Optimal number of clusters for the 26 indices in the main dataset without bipolar disorder patients.


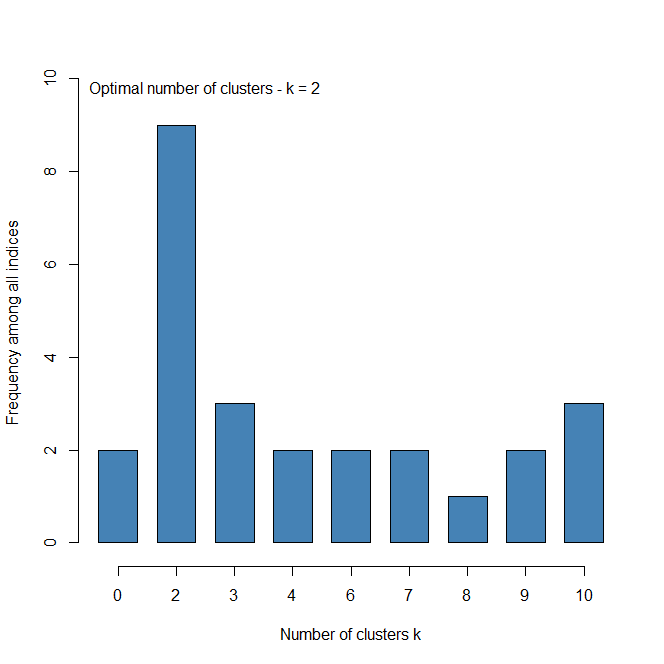


**Figure S6.** Optimal number of clusters for the 26 indices in the replication dataset without bipolar disorder patients.

### **Supplementary Tables**

| **Table S1.** Regional thickness and volumes | | | |
| --- | --- | --- | --- |
|  | Cluster 1  (n = 49) | Cluster 2  (n = 41) | Controls  (n = 49) |
| Regional thickness (mm) |  |  |  |
| Left caudal anterior cingulate | 2.654 (0.209) | 2.559 (0.195)* | 2.681 (0.201) |
| Left caudal middle frontal | 2.481 (0.191) | 2.490 (0.159) | 2.544 (0.132) |
| Left cuneus | 1.900 (0.189) | 1.841 (0.127) | 1.867 (0.122) |
| Left inferior parietal | 2.386 (0.145) | 2.389 (0.150) | 2.427 (0.116) |
| Left medial orbitofrontal | 2.383 (0.156) | 2.363 (0.115) | 2.432 (0.126) |
| Left parahippocampal | 2.859 (0.290) | 2.765 (0.234)** | 2.953 (0.281) |
| Left pars orbitalis | 2.648 (0.241) | 2.581 (0.222)* | 2.720 (0.204) |
| Left pars triangularis | 2.429 (0.180)* | 2.409 (0.159)*** | 2.535 (0.137) |
| Left precentral | 2.544 (0.189)** | 2.559 (0.155)* | 2.643 (0.127) |
| Left rostral anterior cingulate | 2.756 (0.211) | 2.730 (0.198)** | 2.861 (0.196) |
| Left rostral middle frontal | 2.289 (0.145) | 2.259 (0.123) | 2.353 (0.124) |
| Left superior frontal | 2.634 (0.183)* | 2.625 (0.157)* | 2.725 (0.132) |
| Left superior temporal | 2.729 (0.185)** | 2.696 (0.190)*** | 2.842 (0.146) |
| Left insula | 2.958 (0.193) | 2.937 (0.197)* | 3.036 (0.166) |
| Right caudal anterior cingulate | 2.542 (0.218) | 2.445 (0.222)*/# | 2.573 (0.229) |
| Right caudal middle frontal | 2.450 (0.167) | 2.477 (0.162) | 2.531 (0.151) |
| Right cuneus | 1.886 (0.136) | 1.819 (0.130)*/# | 1.884 (0.111) |
| Right inferior parietal | 2.383 (0.136)** | 2.394 (0.125)*** | 2.473 (0.105) |
| Right medial orbitofrontal | 2.431 (0.159) | 2.409 (0.150) | 2.477 (0.114) |
| Right parahippocampal | 2.752 (0.242) | 2.705 (0.223) ** | 2.861 (0.213) |
| Right pars orbitalis | 2.622 (0.179) | 2.547 (0.175)** | 2.668 (0.167) |
| Right pars triangularis | 2.403 (0.179)* | 2.397 (0.173)* | 2.500 (0.154) |
| Right precentral | 2.509 (0.195)* | 2.531 (0.152) | 2.606 (0.120) |
| Right rostral anterior cingulate | 2.905 (0.220) | 2.809 (0.177) | 2.896 (0.198) |
| Right rostral middle frontal | 2.281 (0.135)* | 2.255 (0.134) *** | 2.358 (0.138) |
| Right superior frontal | 2.659 (0.177)* | 2.635 (0.158)** | 2.750 (0.145) |
| Right superior temporal | 2.761 (0.177)*** | 2.746 (0.188)*** | 2.890 (0.157) |
| Right insula | 3.013 (0.187) | 2.920 (0.187)***/# | 3.071 (0.156) |
| Regional volumes (mm^3^) |  |  |  |
| Left thalamus | 7,240.294 (967.068) | 7,156.295 (802.554) | 7,653.596 (851.260) |
| Left caudate | 3,529.412 (580.286) | 3,498.229 (598.958) | 3,408.412 (538.742) |
| Left putamen | 4,932.667 (604.082) | 4,885.737 (730.419) | 4,941.288 (621.422) |
| Left pallidum | 1,975.359 (224.746) | 1,972.212 (309.318) | 1,957.667 (197.212) |
| Left hippocampus | 3,966.618 (474.750) | 3,964.639 (485.384) | 4,123.382 (442.373) |
| Right thalamus | 6,936.996 (880.214) | 6,783.278 (814.356) | 7,239.076 (746.112) |
| Right caudate | 3,609.700 (569.740) | 3,598.261 (613.656) | 3,533.641 (542.152) |
| Right putamen | 4,902.557 (617.950) | 4,848.724 (716.792) | 4,923.053 (616.887) |
| Right pallidum | 1,924.063 (210.197) | 1,932.088 (288.818) | 1,927.304 (207.825) |
| Right hippocampus | 4,057.890 (472.926) | 4,108.310 (469.691) | 4,258.539 (411.257) |
| Data are shown as mean (SD). Only statistically significant results for the ANOVA tests that survive the correction for multiple comparisons are shown.  **p*<0.05; ***p*<0.01; ****p*<0.001 as compared to healthy controls  # *p*<0.05 between patients’ clusters | | | |

**Table S2.** Results of the classification of the main dataset with the k-means and CLARA methods

|  | **CLARA = cluster 1** | **CLARA = cluster 2** |
| --- | --- | --- |
| **k-means = cluster 1** | 75 | 0 |
| **k-means = cluster 2** | 6 | 68 |

**Table S3.** Results of the classification of the main dataset with k-means and the discriminant function

|  | **LDA = cluster 1** | **LDA = cluster 2** |
| --- | --- | --- |
| **k-means = cluster 1** | 72 | 3 |
| **k-means = cluster 2** | 3 | 71 |

LDA = linear discriminant analysis.

**Table S4.** Results of the classification of the replication dataset with k-means and the discriminant function from the main dataset

|  | **k-means = cluster 1** | **k-means = cluster 2** |
| --- | --- | --- |
| **LDA = cluster 1** | 14 | 1 |
| **LDA = cluster 2** | 5 | 14 |

**Table S5.** Coefficients of the linear discriminant function for the analysis without bipolar disorder patients

| **Variable** | **Discriminant coefficient** |
| --- | --- |
| **Pre-stimulus Path Length** | 1.050 |
| **Pre-stimulus Connectivity Strength** | 0.299 |
| **Modulation Path Length** | 1.585 |
| **Modulation Connectivity Strength** | -0.499 |
| **Pre-stimulus Small-worldness** | 0.458 |
| **Modulation Small-worldness** | 3.007 |

**Table S6.** Clinical and cognitive values in FE patients

|  | Cluster 1 (n=17) | Cluster 2 (n=18) |
| --- | --- | --- |
| Age | 29.59 (9.06) | 32.00 (11.53) |
| Parents education (years) | 13.86 (5.30) | 10.90 (2.51) |
| Verbal memory (BACS) | 42.15 (8.72) | 38.94 (7.18) |
| Working memory (BACS) | 19.31 (4.80) | 15.44 (4.00)* |
| Motor speed (BACS) | 61.54 (14.77) | 60.81 (17.50) |
| Verbal fluency (BACS) | 18.50 (8.17) | 18.82 (6.41) |
| Performance speed (BACS) | 48.77 (11.94) | 44.56 (11.70) |
| Problem solving | 17.17 (2.95) | 14.75 (3.71)# |
| Total IQ (WAIS) | 88.23 (14.51) | 87.50 (17.06) |
| % Perseverative errors (WCST) | 14.24 (10.64) | 16.01 (7.29) |
| Emotional intelligence (MSCEIT) | 101.18 (20.45) | 102.43 (18.67) |
| Social Cognition (GEOPTE Scale) | 23.55 (7.15) | 30.88 (12.04) |
| Total negative symptoms (BNSS) | 20.88 (15.61) | 21.38 (17.80) |
| Positive symptoms (PANSS) | 11.29 (3.00) | 10.94 (4.25) |
| Total symptoms (PANSS) | 46.76 (11.33) | 48.31 (13.80) |
| CPZ equivalents (mg/d) | 347.66(204.26) | 325.53(255.19) |

Data are shown as mean (SD).

*p<0.05; #p=0.10

**Table S7.** Functional network values in FE patients

|  | Cluster 1  (n = 17) | Cluster 2  (n = 18) |
| --- | --- | --- |
| Averaged clustering coefficient (CLC) | 1.005 (0.004) | 1.006 (0.003) |
| Characteristic path length (PL) | 1.083 (0.032) | 1.103 (0.028)* |
| Connectivity strength (CS) | 0.310 (0.042) | 0.317 (0.034) |
| CLC modulation | 0.002 (0.001) | 0.000 (0.001)*** |
| PL modulation | 0.010 (0.007) | -0.005 (0.004)*** |
| D modulation | 0.004 (0.006) | 0.000 (0.009) |
| Small-World index | 0.929 (0.025) | 0.913 (0.022)* |
| Small-World modulation | -0.007 (0.005) | 0.004 (0.003)*** |

Data are shown as mean (SD)

*p<0.05; *** p<0.001

### **Supplementary References**

Bachiller, A., Díez, A., Suazo, V., Domínguez, C., Ayuso, M., Hornero, R., … Molina, V. (2014). Decreased spectral entropy modulation in patients with schizophrenia during a P300 task. *European Archives of Psychiatry and Clinical Neuroscience*, *264*(6), 533–543. https://doi.org/10.1007/s00406-014-0488-6

Bledowski, C., Prvulovic, D., Hoechstetter, K., Scherg, M., Wibral, M., Goebel, R., & Linden, D. E. J. (2004). *Behavioral/Systems/Cognitive Localizing P300 Generators in Visual Target and Distractor Processing: A Combined Event-Related Potential and Functional Magnetic Resonance Imaging Study*. https://doi.org/10.1523/JNEUROSCI.1897-04.2004

Gomez-Pilar, J., de Luis-García, R., Lubeiro, A., de la Red, H., Poza, J., Núñez, P., … Molina, V. (2018). Relations between structural and EEG-based graph metrics in healthy controls and schizophrenia patients. *Human Brain Mapping*, *39*(8), 3152–3165. https://doi.org/10.1002/hbm.24066

Gomez-Pilar, J., de Luis-García, R., Lubeiro, A., de Uribe, N., Poza, J., Núñez, P., … Molina, V. (2018). Deficits of entropy modulation in schizophrenia are predicted by functional connectivity strength in the theta band and structural clustering. *NeuroImage: Clinical*, *18*, 382–389. https://doi.org/10.1016/j.nicl.2018.02.005

Núñez, P., Poza, J., Bachiller, A., Gómez-Pilar, J., Lubeiro, A., Molina, V., & Hornero, R. (2017). Exploring Non-Stationarity Patterns in Schizophrenia: Neural Reorganization Abnormalities in the Alpha Band - PubMed. *Journal of Neural Engineering*, *14*(4). Retrieved from https://pubmed.ncbi.nlm.nih.gov/28424430-exploring-non-stationarity-patterns-in-schizophrenia-neural-reorganization-abnormalities-in-the-alpha-band/

Rubinov, M., & Sporns, O. (2010). Complex network measures of brain connectivity: Uses and interpretations. *NeuroImage*, *52*(3), 1059–1069. https://doi.org/10.1016/j.neuroimage.2009.10.003

Scheeringa, R., Fries, P., Petersson, K. M., Oostenveld, R., Grothe, I., Norris, D. G., … Bastiaansen, M. C. M. (2011). Neuronal Dynamics Underlying High- and Low-Frequency EEG Oscillations Contribute Independently to the Human BOLD Signal. *Neuron*, *69*(3), 572–583. https://doi.org/10.1016/j.neuron.2010.11.044

Stam, C. J., De Haan, W., Daffertshofer, A., Jones, B. F., Manshanden, I., Van Cappellen Van Walsum, A. M., … Scheltens, P. (2009). Graph theoretical analysis of magnetoencephalographic functional connectivity in Alzheimer’s disease. *Brain*, *132*(1), 213–224. https://doi.org/10.1093/brain/awn262
